# Supplementary material for: Dynamic transcriptomic profiles of zebrafish gills in response to zinc supplementation
Source: BMC Genomics. 2010 Oct 11;11:553. doi: 10.1186/1471-2164-11-553 (PMC3091702; doi:10.1186/1471-2164-11-553)
Supplement: Additional file 2 — Interactive Direct Interaction Network representing the molecular interactions between zinc, copper, iron, calcium and proteins encoded by transcripts changed by zinc supplementation. Mini web-site containing index.html and hyperlinked pages in subdirectory describing a Direct Interaction Network automatically generated based on curated interactions contained within the proprietary PathwayArchitect database. Ovals represent proteins and the circles symbolize metal ions. Objects are coloured by their abundance in zebrafish at the time-point they were significantly different from the control is a scale from -4 fold (dark green) to +4 fold (dark red). Where significant differences were found at more than one time-point, the colour overlay shows expression at the first instance. Dark blue squares denote 'binding', and light blue squares 'expression'; green squares stand for 'regulation', green diamonds for 'metabolism', and green circles for 'promoter binding'. Arrow heads indicate directionality of the interaction where annotated. All nodes and edges can be further interrogated by selecting the relative area of the image. [file 1471-2164-11-553-S2.zip › PathwayArchitect Zn xs DIN/148187.html]

# PROTEIN: TCF8

|  |  |
| --- | --- |
| Name | TCF8 |
| Type | PROTEIN |
| Description | transcription factor 8 (represses interleukin 2 expression) |
| Note | TCF8 encodes a human zinc finger transcription factor that represses T-lymphocyte-specific IL2 gene (MIM 147680) expression by binding to a negative regulatory domain 100 nucleotides 5-prime of the IL2 transcription start site (Williams et al., 1991).[supplied by OMIM] |
| Alias | Zinc finger homeodomain enhancer-binding protein |
|  | zinc finger homeodomain enhancer-binding protein |
|  | Zfhx1a |
|  | NIL-2A |
|  | transcription factor 8 (represses interleukin 2 expression) (TCF8) |
|  | transcription factor 8 |
|  | ZEB1 |
|  | ZFHEP |
|  | ZEB |
|  | Zfhep |
|  | BZP |
|  | NIL-2-A |
|  | AREB6 |
|  | deltaEF1 |
|  | Negative regulator of IL2 |
|  | Tcf8 |
|  | TCF8 |
|  | ZFHX1A |
|  | NIL-2-A zinc finger protein |


---

|  |  |
| --- | --- |
| GO Component | nucleus |


---

|  |  |
| --- | --- |
| GO ID | GO:0005634 |
|  | GO:0003677 |
|  | GO:0003714 |
|  | GO:0006355 |
|  | GO:0003690 |
|  | GO:0003676 |
|  | GO:0000122 |
|  | GO:0046872 |
|  | GO:0003713 |
|  | GO:0008283 |
|  | GO:0006955 |
|  | GO:0008270 |
|  | GO:0003700 |


---

|  |  |
| --- | --- |
| MIM | MIM:189909 |


---

|  |  |
| --- | --- |
| Connectivity | 92 |


---

|  |  |
| --- | --- |
| Entrez ID | 6935 |
|  | 25705 |


---

|  |  |
| --- | --- |
| Agilent ID | A\_23\_P202010 |
|  | A\_44\_P153979 |
|  | A\_42\_P505798 |
|  | A\_14\_P116868 |
|  | A\_23\_P202013 |
|  | A\_14\_P102704 |
|  | A\_14\_P111969 |
|  | A\_14\_P102626 |
|  | A\_14\_P118603 |
|  | A\_14\_P129718 |


---

|  |  |
| --- | --- |
| Cellular Localization | Nucleus |
|  | Organelle |
|  | Cell |


---

|  |  |
| --- | --- |
| Pathway | Zn xs inventory |
|  | Zn xs DIN |


---

|  |  |
| --- | --- |
| GO Process | cell proliferation |
|  | immune response |
|  | negative regulation of transcription from RNA polymerase II promoter |
|  | regulation of transcription, DNA-dependent |


---

|  |  |
| --- | --- |
| UniGene | Rn.10600 |
|  | Hs.124503 |


---

|  |  |
| --- | --- |
| Affymetrix Probeset ID | 1370965\_at |
|  | 1376673\_at |
|  | 1380200\_at |
|  | 210875\_s\_at |
|  | 212758\_s\_at |
|  | 57311\_at |
|  | 58210\_at |
|  | 74451\_at |
|  | 1384947\_at |
|  | g529172\_3p\_a\_at |
|  | Hs.232068.2.A1\_3p\_s\_at |
|  | rc\_AI102906\_at |
|  | U51583\_at |
|  | U51584\_s\_at |
|  | RC\_AA371432\_at |
|  | RC\_AA406078\_at |
|  | rc\_AA956592\_at |


---

|  |  |
| --- | --- |
| GO Function | transcription coactivator activity |
|  | double-stranded DNA binding |
|  | transcription factor activity |
|  | transcription corepressor activity |
|  | DNA binding |
|  | zinc ion binding |
|  | nucleic acid binding |
|  | metal ion binding |


---

|  |  |
| --- | --- |
| Nucleotide | AL117340 |
|  | M81699 |
|  | AY325179 |
|  | NM\_030751 |
|  | AL161935 |
|  | AL158080 |
|  | U12170 |
|  | AL355148 |
|  | AL831979 |
|  | NM\_013164 |
|  | AK091478 |
|  | BX647794 |
|  | U51583 |


---

|  |  |
| --- | --- |
| Protein | NP\_110378 |
|  | CAI17321 |
|  | AAP92580 |
|  | CAI15108 |
|  | NP\_037296 |
|  | Q62947 |
|  | CAI12549 |
|  | CAH74131 |
|  | CAI15107 |
|  | CAI12550 |
|  | AAB17130 |
|  | CAI17319 |
|  | CAI15109 |
|  | BAC03673 |
|  | CAH74132 |
|  | CAI12551 |
|  | P37275 |
|  | AAA20602 |
|  | CAI17320 |


---

|  |  |
| --- | --- |
| Organism | Mammal |


---

|  |  |
| --- | --- |
| Location | chromosome 17, 17q12.1 (Rattus norvegicus) |
|  | chromosome 10, 10p11.2 (Homo sapiens) |


---

|  |  |
| --- | --- |
